# Traumatic coccydynia patients benefit from coccygectomy more than patients undergoing coccygectomy for non-traumatic causes

**DOI:** 10.1186/s13018-023-04098-5

**Published:** 2023-10-27

**Authors:** Deniz Kara, Anil Pulatkan, Vahdet Ucan, Said Orujov, Mehmet Elmadag

**Affiliations:** 1 Orthopaedic Department, Washington University School of Medicine, Saint Louis, MO USA; 2 Department of Orthopedics and Traumatology, Bezmialem Vakif University School of Medicine, Fatih, Istanbul, Turkey

**Keywords:** Coccyx, Coccydynia, Coccygectomy, Etiology, Tail bone

## Abstract

**Purpose:**

Conservative treatment is the first step in the management of coccydynia. However, surgical treatment is required in cases where conservative treatment fails. The aim of this study was to compare the effect of traumatic and atraumatic etiologies on functional outcomes in patients who underwent coccygectomy for chronic coccydynia.

**Methods:**

Ninety-seven patients who underwent partial coccygectomy between October 2010 and December 2018 for the diagnosis of chronic coccygodynia were evaluated retrospectively. The patients were divided into two groups according to etiologies as atraumatic (group AT) and traumatic (group T). Concomitant disorders of the patients were recorded as psychiatric and musculoskeletal diseases. Visual Analog Scale (VAS) for low back pain, the Oswestry Disability Index (ODI) scale, Short Form-36 Physical Component Summary and Short Form-36 Mental Component Summary were used to evaluate the clinical outcomes pre- and postoperative at the last follow-up.

**Results:**

The mean follow-up time was 67.3 ± 13.9 (range; 44–115) months. Group AT and group T included 48 (mean age 37.1 ± 11.3 and 36 (75%) female) and 49 patients (mean age 36 ± 11 and 35 (71.4%) female), respectively. The groups were statistically similar in terms of age (*p* = 0.614), gender (*p* = 0.691), body mass index (*p* = 0.885), tobacco usage (*p* = 0.603) and duration of pain (*p* = 0.073). However, the rate of musculoskeletal and total concomitant disorders was higher in the Group AT than in Group T (*p* < 0.05). The average preoperative SF-36 MCS and SF-36 PCS scores improved at the last follow-up from 43.3 ± 6.2 and 35.6 ± 4.9 to 72 ± 14.1 and 58.3 ± 10.9, respectively. The preoperative VAS and ODI decreased from 8 ± 1.4 and 39.8 ± 8.5 to 2.6 ± 1.8 and 13.4 ± 8.9 at the last follow-up, respectively.

**Conclusion:**

Successful results were obtained with surgical treatment in chronic coccygodynia. In addition, functional outcomes in patients with traumatic etiology are better than in atraumatic ones.

*Levels of evidence* Level III; Retrospective Comparative Study.

## Introduction

Coccydynia defined as the painful condition of the coccygeal region, especially when sitting [1]. The most common cause of coccydynia is trauma to the coccyx with axial loading [2]. In addition, sitting on a hard, narrow or uncomfortable surface for a long time, chordoma, intradural mass, infection, coccyx degeneration, difficult vaginal delivery, sudden weight loss and neurotic disorders are other known etiologies [3, 4].

The first stage in the treatment of coccydynia is conservative modalities, including non-steroidal anti-inflammatory drugs, ring-shaped cushions, hot water baths, local corticosteroid or platelet-rich plasma injections, massage, radiofrequency denervation, shock wave therapy and ganglion impar blocks [4–10]. Coccygectomy is performed with successful outcomes in patients with chronic coccydynia for whom conservative treatments have failed [11–16]. Although the classical literature shows that surgical treatment of traumatic coccydynia is more successful than those of atraumatic patients, there are recent studies showing that there is no difference between traumatic and atraumatic patients in terms of functional outcomes, and the literature is controversial on this issue [12, 17–22].

The aim of this study was to compare the effect of traumatic and atraumatic etiologies on functional outcomes in patients who underwent coccygectomy for chronic coccydynia. Our hypothesis was that the functional outcomes of the patients who underwent coccygectomy for traumatic etiologies were superior than that who underwent coccygectomy for atraumatic etiologies.

## Methods

This single-center comparative retrospective study was conducted in Bezmialem Vakıf Univeristy Department of Orthopedics and Traumatology between October 2010 to December 2018. The study approval was taken from the local institutional review board. All patients voluntarily participated in the study after receiving an explanation of the risks and benefits and were required to sign an informed consent form prior to enrollment. A structured questionnaire was used to gather information on the following patient metrics: descriptive data, concomitant disorders, American Society of Anesthesiologists (ASA) physical status classification [23], Short Form 36 Physical Component Summary (SF-36 PCS) and Short Form 36 Mental Component Summary (SF-36 MCS) [24], the Oswestry Disability Index (ODI) [25, 26], a Visual Analog Scale (VAS) for pain, preoperative duration of pain, postoperative follow-up times and complication rate. The term traumatic coccydynia was defined as injury to the coccyx after mechanical loading on the coccyx. Patients who underwent coccygectomy for traumatic and atraumatic etiologies were compared with each other in terms of these data. Concomitant disorders of the patients were recorded as psychiatric and musculoskeletal diseases. Major depression disorder, anxiety disorder, bipolar disorder, post-traumatic stress disorder, attention deficit-hyperactivity disorder and obsessive-compulsive disorder defined as psychiatric concomitant disorders and lumbar spine pathology, fibromyalgia and chronic pain syndrome defined as musculoskeletal concomitant disorders.

### Patient selection

Specific tenderness was detected in all patients with bimanual palpation by an experienced surgeon in the coccygeal region. Perianal soft tissue pathologies including anal fistulas, anal fissures and hemorrhoids were excluded in all patients by careful clinical assessment. All patients were examined with 2-way lumbosacral, coccyx radiographs and magnetic resonance images (MRI). Surgery was indicated for patients who (1) were coccygeal flexion angle greater than 25 degrees with direct axial loading on the lateral radiograph [27] (2) had coccydynia for at least 6 months that did not respond to conservative treatment and (3) were administered local corticosteroid injections (40 mg methylprednisolone acetate 20 mg/mL, 1 cc prilocaine HCl) to the coccygeal region at least 2 times with an interval of 2 to 3 months regardless of the result of radiological imaging. Patients older than 18 years of age and followed for at least 2 years with enough documentation (medical record and informed consent) were included in the study.

Patients under of 18 years of age, who had previous coccyx surgery for any reason including trauma, tumor, abscess and infection, who had suspected tumor or infection according to the MRI findings, lost to follow-up and without enough documentation were excluded from the study.

The hospital records of a total of 689 coccygodynia patients were reviewed, and 170 of these had chronic coccygodynia. A total of 97 patients with chronic coccygodynia, who underwent partial coccygectomy because of chronic coccygodynia, were included in the study, and all patients included in the study had similar surgical indications.

### Surgical technique

**Fig. 1:**
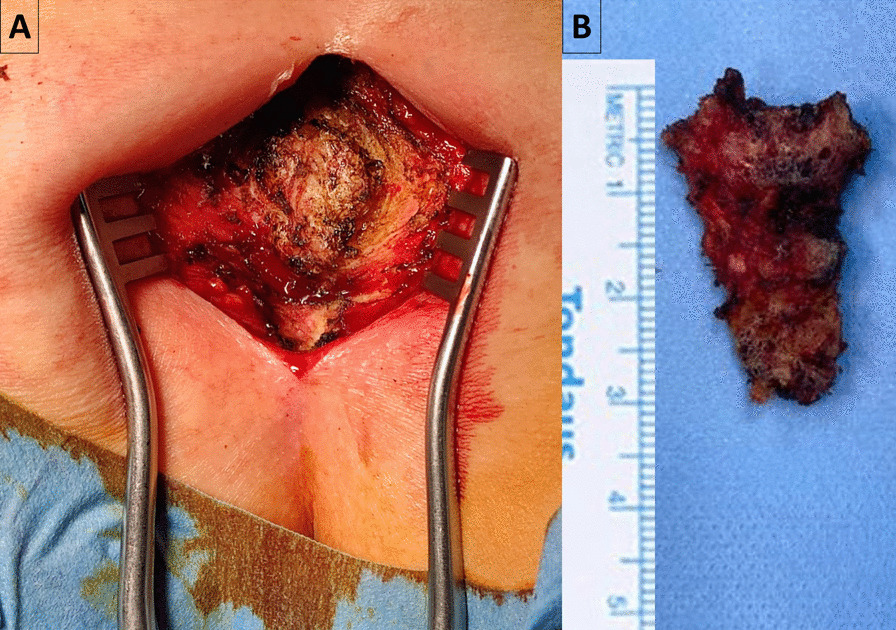
**A** Intraoperative photograph of a 32-year-old patient with a traumatic coccyx instability, **B** Bone fragment removed by partial coccygectomy

All operations were performed by one senior spine surgeon using the same technique. All patients received 2 g cefazolin intravenous (IV) antibiotic prophylaxis preoperatively. The partial coccygectomy procedure was performed under general or spinal anesthesia. The patients were placed prone, and the procedure was similar to the technique, which described by Key [28]. A 6 cm longitudinal incision was made from the midline of the sacrococcygeal region to 4 cm away from the anus. Posterior surface of coccyx was exposed subperiosteally using cauterization. After the unstable part exposed, it was carefully separated from the anterior soft tissue and resected. Hemostasis was performed, and then, all layers were closed with special attention to avoid any gaps, and no drains were used in any patient (Fig. 1).

### Postoperative follow-up protocol and functional assessment

A standard postoperative rehabilitation protocol was applied for all patients. 1 g of cefazolin was administered every 8 h for one day after surgery. The initial postoperative mobilization was performed under the supervision of the physiotherapist. The patients were advised to use a ring-shaped cushing for 4 weeks postoperatively. A postoperative laxative or enema was administered on for postoperative three days to prevent the pain and constipation. In addition, patients were advised not to lift heavy things and to nutrition with dietary fiber for three months. Routine follow-up was performed at 2 weeks, 6 weeks, 3 months, 6 months, 1 year, and annually thereafter.

The functional assessments were performed by a senior physiotherapist, who was blinded to the study groups pre- and postoperative at the last follow-up. The improvements in the functional scores were noted as delta scores. In addition, all patients were categorized according to the success of the treatment. The threshold for successful treatment was based on a 20-point minimum difference in clinical significance (MCID) from baseline to last follow-up on the ODI [25, 29]. Those who did not meet this threshold were classified as treatment failure.

### Statistical analysis

SPSS 20.0 statistical package software (IBM, Armonk, NY, USA) was used to analyze the collected data. The normal distribution was tested by the Shapiro–Wilk test. The group comparisons were performed using the independent samples *t*-test and Mann–Whitney U test. For categorical data, the Chi-square test was used. Data were given as mean ± standard deviation with medians and ranges. Categorical variables were given with frequency (percentage) values. The significance level was determined at a *p* value of < 0.05.

## Results

### Patient demographics

**Fig. 2:**
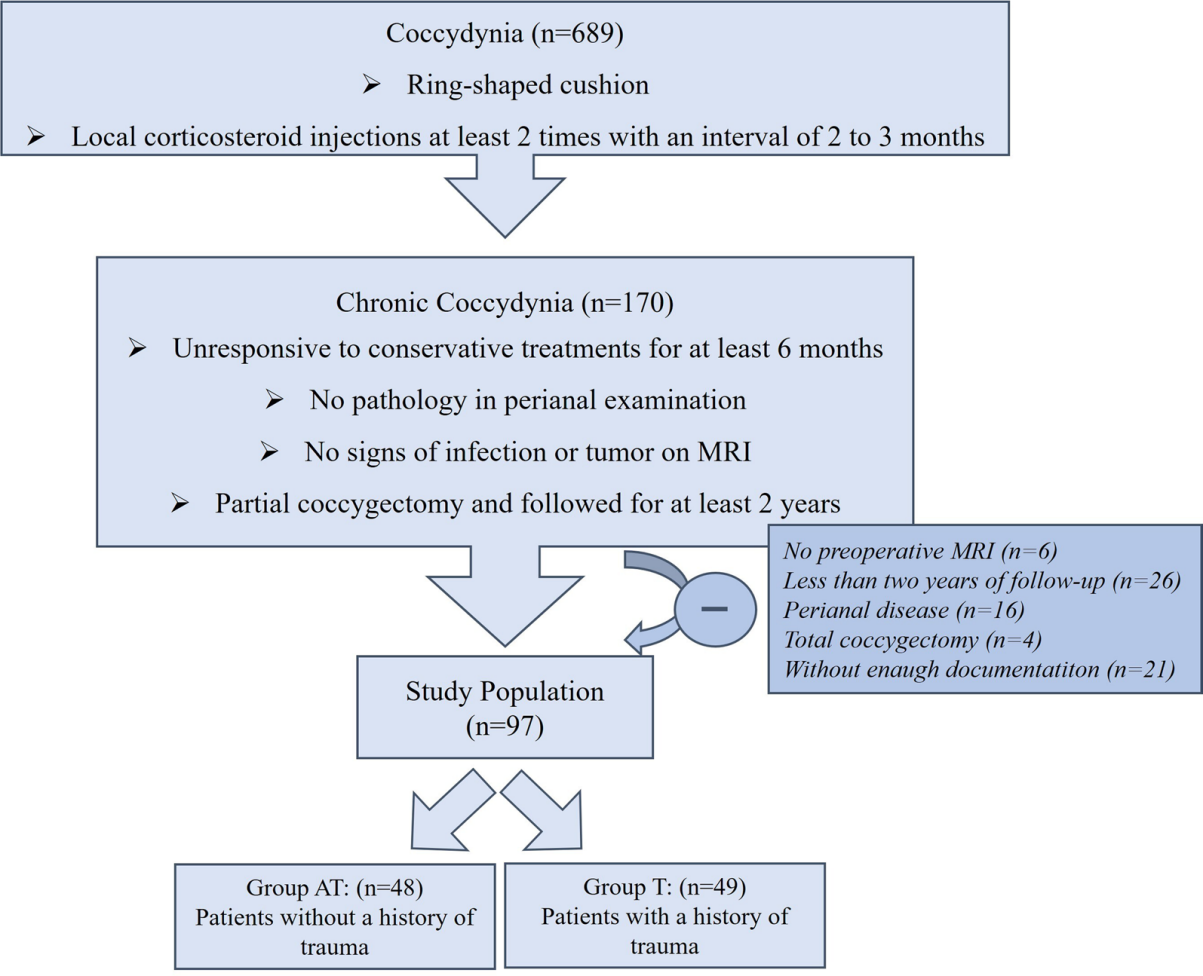
Study flowchart showing inclusion and exclusion steps

Among the patients who underwent treatment with the partial coccygectomy for chronic coccygodynia, 97 met the inclusion criteria. The patients were divided into two groups according to history of trauma: the post-traumatic (Group T) and the atraumatic (Group AT) (Fig. 2). The mean age of the patients was 36.5 ± 11.1 years. Regarding gender, 73% (*n* = 71) of the patients were female and 27% (*n* = 26) were male. The average follow-up for all patients is 67.3 ± 13.9 (range; 44–115) months after surgery. The mean BMI of patients was 27.2 ± 4.7 kg/m^2^. The patients had pain for an average of 23 ± 15.9 (range; 6–96) months before the operation. The demographics of the patients were similar in both groups (Table 1). On the other hand, the rate of musculoskeletal (20/48 vs. 7/49; *p* = 0.002) and total concomitant disorders (30/48 vs. 12/49; *p* = 0.001) was found to be significantly higher in group AT than in group T (Table 2).

**Table 1:** Patient demographics

	Group AT (*n* = 48)	Group T (*n* = 49)	*p* values
	Descriptive statistics		
Age (Years)	37.1 ± 11.3	36 ± 11	0.614^a^
Gender (Female) (%)	36 (75%)	35 (71%)	0.691^b^
Follow-up (months)	64.4 ± 12.8 [64:44–96]	70 ± 14.5 [65:47–115]	0.068^c^
BMI (kg/m^2^)	27.1 ± 3.7	27.2 ± 4	0.885^a^
Tobacco use (%)	14 (29%)	12 (25%)	0.603^b^
Duration of pain (months)	22.1 ± 19.1 [12:6–96]	23.9 ± 12 [24:8–48]	0.073^c^
ASA	1.3 ± 0.5 [1:1–3]	1.2 ± 0.4 [1:1–3]	0.503^c^

ASA: American Society of Anesthesiologists; BMI: Body Mass Index

^a^*t*-test

^b^Chi-square test

^c^Mann Whitney test

**Table 2:** Demographics of concomitant disorders between the groups

	Group AT (*n* = 48)	Group T (*n* = 49)	*p* values
	Descriptive statistics		
Musculoskeletal (%)	20 (42%)	7 (14%)	0.002^a^
Spondyloarthropathy	1 (2%)	1 (2%)	
Musculoskeletal pain	5 (10%)	1 (2%)	
Fibromyalgia	7 (14%)	2 (4%)	
Chronic pain syndrome	6 (13%)	2 (4%)	
Lumbar spine pathology	1 (2%)	1 (2%)	
Psychiatric (%)	10 (21%)	5 (10%)	0.147^a^
Major depression disorder	3 (6%)	1 (2%)	
Anxiety disorder	3 (6%)	1 (2%)	
Bipolar disorder	1 (2%)	1 (2%)	
Post-traumatic stress disorder	1 (2%)	2 (4%)	
Obsessive-compulsive disorder	2 (4%)	–	
Total concomitant disorders (%)	30 (63%)	12 (24%)	0.001^a^

^a^Chi-square test

The most common etiology of coccygodynia was idiopathic causes (48%; *n* = 23), coccygeal spicule (23%; *n* = 11), weight loss (25%; *n* = 12), and recent lumbar spine surgery (4%; *n* = 2) in group AT.

#### Functional outcomes

The average preoperative SF-36 MCS and SF-36 PCS scores improved at the last follow-up from 43.3 ± 6.2 and 35.6 ± 4.9 to 72 ± 14.1 and 58.3 ± 10.9, respectively. The preoperative VAS and ODI decreased from 8 ± 1.4 and 39.8 ± 8.5 to 2.6 ± 1.8 and 13.4 ± 8.9 at the last follow-up, respectively (Table 3).

**Table 3:** Functional outcomes between the groups

	Group AT (*n* = 48)	Group T (*n* = 49)	*p* values
	Descriptive statistics		
Preoperative ODI	40.9 ± 8.8 [42:10–64]	38.8 ± 8.1 [38:24–56]	0.134^a^
Postoperative ODI	15.6 ± 9.9 [12:6–38]	11.1 ± 7.3 [10:4–34]	**0.004**^**a**^
ΔODI	25.3 ± 10.9 [28:0–48]	27.7 ± 10.8 [28:2–48]	0.397^a^
Preoperative SF-36 MCS	43.8 ± 5.2 [44:30–56]	42.8 ± 7.1 [42:32–65]	0.08^a^
Postoperative SF-36 MCS	68.3 ± 16 [72:35–89]	75.7 ± 10.9 [79:40–91]	**0.014**^**a**^
ΔSF-36 MCS	24.5 ± 15.5 [26.5:-4–50]	32.8 ± 12.2 [33:3–53]	**0.003**^**a**^
Preoperative SF-36 PCS	36 ± 5.1 [36:28–48]	35.1 ± 4.7 [35:28–48]	0.291^a^
Postoperative SF-36 PCS	56.5 ± 12.9 [58:34–76]	60 ± 8.4 [59:41–80]	0.293^a^
ΔSF-36 PCS	20.4 ± 13.4 [20.5:-3–45]	24.9 ± 10.2 [27:0–46]	0.092^a^
Preoperative VAS	8 ± 1.3 [8:6–10]	8.1 ± 1.5 [8:5–10]	0.631^a^
Postoperative VAS	3.2 ± 1.9 [3:0–8]	2 ± 1.3 [2:0–6]	**0.001**^**a**^
ΔVAS	4.8 ± 2.3 [4:1–10]	6.1 ± 2.4 [6:0–10]	**0.006**^**a**^
Treatment success (%)	34/48 (71%)	43/49 (88%)	**0.047**^**b**^
Complication (%)	8/48 (17%)	7/49 (14%)	0.746^b^

Bold: *p* < 0.05

ODI: Oswestry Disability Index; SF-36 MCS: Short Form 36 Mental Component Summary; SF-36 PCS: Short Form 36 Physical Component Summary; VAS: Visual Analog Scale

^a^Mann Whitney test

^b^Pearson Chi-Square

There was a significant difference in favor of Group T between the groups in terms of treatment success (*p* = 0.047), postoperative ODI (*p* = 0.004), SF-36 MCS (*p* = 0.014), and VAS (*p* = 0.001), ΔSF-36 MCS (p = 0.003) and ΔVAS (*p* = 0.006) scores.

Post hoc power analysis according to the primary outcomes (ODI, VAS, SF-36 MCS and SF-36 PCS) with a significance level of 0.05 showed a large effect size (2.11) and a power (1-b) between 0.72 and 0.95, which confirmed that the sample size was adequate.

### Complications

Postoperative prolonged drainage was observed in 15/97 (15%) of the patients, and 7 patients recovered with wound care and oral antibiotic therapy (Table 4). A total of 8 patients, 5 from the group AT and 3 from the group T, required further surgery. Escherichia coli was detected in 3 patients, methicillin-sensitive Staphylococcus aureus was detected in 1 patient, and Enterococcus faecalis was detected in 1 patient. These patients recovered with debridement and bacteria-specific antibiotics. The remaining three patients had wound dehiscence alone without infection. Wound healing was achieved with postoperative vacuum assisted closure in these patients at 2 months (Table 4).

**Table 4:** Comparison of demographic data and functional outcomes of infected patients

	Group AT (*n* = 8)	Group T (*n* = 7)	*p* values
	Descriptive statistics		
Age (Years)	35.5 ± 11.1	35 ± 9	0.929^a^
Gender (Female) (%)	6 (75%)	6 (85%)	0.119^b^
Follow-up (months)	63.8 ± 10.8	72 ± 16	0.397^c^
BMI (kg/m^2^)	28.3 ± 4.2	28.4 ± 3	0.922^a^
Tobacco use (%)	2 (25%)	1 (15%)	1^b^
Duration of pain (months)	28 ± 6	40 ± 31	0.855^c^
Preoperative ODI	37.8 ± 5.5	36.8 ± 7.1	1^c^
Postoperative ODI	21 ± 11	15.7 ± 12	0.281^c^
Preoperative SF-36 PCS	36.5 ± 6.6	38.7 ± 4.3	0.463^c^
Postoperative SF-36 PCS	53.1 ± 15.5	60.1 ± 10.3	0.613^c^
Preoperative SF-36 MCS	44.8 ± 4.8	42.1 ± 4	0.040^c^
Postoperative SF-36 MCS	60.3 ± 21	65.5 ± 15	0.694^c^
Preoperative VAS	7.5 ± 1.9	7.7 ± 1.5	0.779^c^
Postoperative VAS	3.6 ± 2.1	3.4 ± 1.6	0.867^c^
Treatment success (%)	50% (4/8)	56% (4/7)	1^b^

*BMI* body mass index

^a^*t*-test

^b^Fisher’s Exact test

^c^Mann Whitney test

## Discussion

The significant finding of our study is that patients who underwent partial coccygectomy for chronic coccydynia have high functional results and satisfaction rates. In the comparison of patients who underwent partial coccygectomy for traumatic and atraumatic etiologies, postoperative ODI (11.1 vs. 15.6; *p* = 0.004), VAS (2 vs. 3.2; *p* = 0.001) and SF-36 MCS (75.7 vs. 68.3; *p* = 0.014) were superior in patients with traumatic coccydynia compared to atraumatic patients. In addition, it was retrospectively observed that the rate of musculoskeletal and total concomitant disorders was higher in atraumatic patients than in traumatic ones. Therefore, other sources of pain should be evaluated, and more attention should be paid when deciding on surgery in atraumatic patients.

There is still no consensus in the literature in the comparison of the outcomes of coccygectomy performed for atraumatic and traumatic etiologies. Pennekamp et al. [12] compared the results of patients who underwent coccygectomy for 8 traumatic and 8 atraumatic etiologies and presented mean follow-up of 7.3 years. They reported good and excellent results in 7 (88%) patients who underwent coccygectomy for traumatic reasons and 3 (38%) patients who underwent coccygectomy for atraumatic ones. Similarly, Cebesoy et al. [19] compared the results of patients who underwent coccygectomy for traumatic and atraumatic etiologies and were followed up for at least 2 years. The traumatic coccydynia group showed good results with a 75% success rate, which was followed by the idiopathic spontaneous group with a 58% success rate. However, Kerr et al. presented the results of 62 patients who underwent coccygectomy and no significant difference in outcome could be detected based on traumatic versus non-traumatic causes [17]. In our study, the functional results of 48 atraumatic and 49 traumatic patients with an average follow-up of 67 months were compared; postoperative VAS, ODI, and SF-36 MCS results were found to be significantly superior in the traumatic group.

Concomitant disorder may cause poor health-related quality of life and functional outcomes following coccygectomy. Hanley et al. reported the 2-year results of 98 coccygectomy patients in a prospective study, comparing successful and failed outcomes according to the MCID on ODI score [30]. They showed in 69 patients successful results and in 25 patients failed results. They found that psychiatric diseases, preoperative opioid use and comorbidity rates were significantly higher in failed outcomes compared to successful patients. Sagoo showed in a review study that included 826 coccygectomy patients that preoperative determination of psychiatric comorbidities would guide the postoperative results [31]. In current study, the rate of musculoskeletal and total concomitant disorders was found to be significantly higher in the atraumatic group than in the traumatic group. This situation may be a result of that comorbidities are more common in atraumatic coccydynia patients compared to traumatic ones. Although concomitant psychiatric disorders were observed twice as often in the atraumatic group as in the traumatic group, there was no significant difference between the two groups because of the low number of concomitant psychiatric patients. Examination of this issue in future studies will guide the estimation of the results of this surgery.

Patient scoring systems have gained importance with the shift of patient-reported outcome measure (PROM) scores regarding health system to the value-based system [32]. MCID is being a popular approach to determine a meaningful improvement in a patient’s condition following a treatment in clinical studies [32, 33].This definition is made to clarify clinically significant improvements in the treatment of patients regardless of statistically significant difference [34]. In our study, similar to previous studies, a change of minimum 20 points in the ODI score after coccygectomy was considered as a successful result and otherwise as a failure of the treatment. Successful treatment results were obtained in a total of 77/97 (79%) patients, 43/49 (88%) from group T and 34/48 (71%) from group AT. This rate is within the range of 68–92% successful outcome after coccygectomy in the literature [13, 35–37]. This definition will become more popular in clinical studies once problems with the heterogeneity and complexity of this definition are resolved.

The surgical site infection is the most common complication after coccygectomy. The rate of infection after coccygectomy is reported up to 27% in the literature [12–15, 17–20, 38]. Sagoo et al. reported in a recent systematic review of 826 coccygectomy patients that the infection rate after coccygectomy was 8%, and the rate of reoperation was 3% [31]. However, Cebesoy et al. [19] reported in their study that there was no infection in 21 patients, who underwent coccygectomy and were administered prophylactic antibiotics for 5 days. Similarly, Doursounian et al. [38] reported that postoperative infection did not develop in patients who were administered two different IV prophylactic antibiotics (cefamandole and ornidazole) for 48 h. They concluded that the improvements in intraoperative wound care and prophylactic intravenous antibiotic administration have reduced surgical site infections. In addition, there are also studies reporting the infection rates of incision techniques. Kulkarni et al. [39] analyzed 10 coccydynia patients who were treated using the Z plasty technique in the coccygectomy and no infection was reported in the patients. Nagappa et al. [40] evaluated 45 patients who underwent treatment with the coccygectomy using the paramedian incision for coccydynia. They concluded that the wound infections were 11% (5 patients). In our study, postoperative prolonged wound drainage was observed in 15/97 patients, and all patients performed a midline incision. Empirical oral antibiotic therapy and wound care were applied to all of these patients without taking a swab culture. Irrigation and debridement were performed to 8/15 patients in the operating room, whose prolonged wound drainage did not resolve. We are in the opinion that the reason of the complication rate is at the upper limit of the literature is that IV antibiotics were administered only postoperative one day and oral or IV antibiotics were not given afterward. Future studies comparing postoperative antibiotic treatment regimens and incision techniques will shed light on the literature and will reduce the complication rates.

This study has several limitations. First, the study was designed retrospectively, and the number of patients was relatively small. Second, similar to the studies in the literature comparing traumatic-atraumatic coccydynia, the number of comorbidities was higher in the atraumatic patient group than in the traumatic group. Third, although all patients had preoperative radiological evaluation, no comparison was performed between the groups in terms of radiological examinations. Fourth, the follow-up period reflects the mid-term outcomes after surgery. Fifth, per-operative data and complications were not used. Sixth, retrolisthesis on lateral x-ray was not evaluated for early surgery. Finally, the number of patients included in the study is not sufficient to reach a clear conclusion regarding the comparison of the groups in terms of complication rate and concomitant psychiatric disorders.

## Conclusion

Good functional results were obtained after partial coccygectomy in both traumatic and atraumatic patients in the treatment of chronic coccydynia. However, traumatic patients have higher satisfaction levels and functional outcomes. Future prospective, randomized, controlled comparative studies involving more patients and with longer follow-up times are needed to show the effect of concomitant disorders on surgical success in coccygectomy surgery.

## Data Availability

The datasets generated during and/or analyzed during the current study are available from the corresponding author on reasonable request.
